# Increased Long-Term Cardiovascular Risk After Total Hip Arthroplasty: A Nationwide Cohort Study: Erratum

**DOI:** 10.1097/01.md.0000484043.51237.3b

**Published:** 2016-05-20

In the article “Increased Long-Term Cardiovascular Risk After Total Hip Arthroplasty: A Nationwide Cohort Study”,^1^ which appeared in Volume 95, Issue 6 of *Medicine*, an incorrect version of Figure 1 originally appeared. The article has since been corrected online.
